# Metabolic Regulation of Carotenoid-Enriched Golden Rice Line

**DOI:** 10.3389/fpls.2016.01622

**Published:** 2016-10-28

**Authors:** Dipak Gayen, Subhrajyoti Ghosh, Soumitra Paul, Sailendra N. Sarkar, Swapan K. Datta, Karabi Datta

**Affiliations:** ^1^Laboratory for Translational Research on Transgenic Crops, Department of Botany, University of CalcuttaKolkata, India; ^2^National Institute of Plant Genome ResearchNew Delhi, India; ^3^Department of Crop Sciences, Institute of Agriculture, Visva Bharati UniversitySantiniketan, India

**Keywords:** golden rice, vitamin A deficiency, substantial equivalence, proteomics, metabolomics

## Abstract

Vitamin A deficiency (VAD) is the leading cause of blindness among children and is associated with high risk of maternal mortality. In order to enhance the bioavailability of vitamin A, high carotenoid transgenic golden rice has been developed by manipulating enzymes, such as phytoene synthase (*psy)* and phytoene desaturase (*crtI*). In this study, proteome and metabolite analyses were carried out to comprehend metabolic regulation and adaptation of transgenic golden rice after the manipulation of endosperm specific carotenoid pathways. The main alteration was observed in carbohydrate metabolism pathways of the transgenic seeds. The 2D based proteomic studies demonstrated that carbohydrate metabolism-related enzymes, such as pullulanase, UDP-glucose pyrophosphorylase, and glucose-1-phosphate adenylyltransferase, were primarily up-regulated in transgenic rice seeds. In addition, the enzyme PPDK was also elevated in transgenic seeds thus enhancing pyruvate biosynthesis, which is the precursor in the carotenoids biosynthetic pathway. GC-MS based metabolite profiling demonstrated an increase in the levels of glyceric acid, fructo-furanose, and galactose, while decrease in galactonic acid and gentiobiose in the transgenic rice compared to WT. It is noteworthy to mention that the carotenoid content, especially β-carotene level in transgenic rice (4.3 μg/g) was significantly enhanced. The present study highlights the metabolic adaptation process of a transgenic golden rice line (homozygous T4 progeny of SKBR-244) after enhancing carotenoid biosynthesis. The presented information would be helpful in the development of crops enriched in carotenoids by expressing metabolic flux of pyruvate biosynthesis.

## Introduction

Carotenoids not only have a high nutritional value but also play a pivotal role in physiological functions such as vision, growth, reproduction, cellular differentiation, proliferation, and immunity (Tang et al., 2009; Wurtzel et al., 2012). Since, carotenoids, including β-carotene, have anti-oxidant properties, they help combat numerous ROS-generated diseases such as cardiovascular diseases, various types of cancer, neurological disorders, photosensitivity, and eye-related disorders (Tang et al., 2009; Farré et al., 2011). Animals cannot synthesize carotenoids, therefore, they need to depend on plant-derived products to meet the requirements for physiological functions.

Worldwide, approximately 250 million pre-school children become blind every year due to vitamin A deficiency (VAD), and 10% of these children die due to increased susceptibility to infectious diseases (Krishnan et al., 2009; Brown and Noelle, 2015).

Transgenic golden rice was initially developed by *Agrobacterium* -mediated transformation of β-carotene biosynthetic pathways into rice endosperm (Ye et al., 2000). Modern advancements in the field of biotechnology have enabled the development of genetically modified (GM) crops overexpressing provitamin A. Such crops include rice (Datta et al., 2003, 2014; Paine et al., 2005; Parkhi et al., 2005; Schaub et al., 2005; Gayen et al., 2015), potato (Römer et al., 2002), tomato (Fraser et al., 2009), canola (Shewmaker et al., 1999) and maize (Zhu et al., 2008). A recent study has reported that GM crops have covered approximately 160 million hectares of land over 29 countries (Khush et al., 2012). It is perceived by some people that genetic manipulation of plants may have some negative effects on the core metabolic pathways resulting in competition for particular precursors, which are also involved in other closely related pathways (Sandmann, 2001). The genetic modifications may lead to intended or unintended modifications. The transgenic plant generally developed by the insertion of a modified T-DNA sequence from *Agrobacterium tumefaciens* or other vector DNA sequences into the genome, which may potentially disrupt the function of native genes and can create rearrangements at the site of insertion. In some instances, somaclonal variation, and pleiotropy may be responsible for unintended effects of transgenic plant (Schnell et al., 2015). Therefore, molecular assessment of transgenic plant as well as agronomic studies is essential for biosafety assessment. However, unintended effects of transgenic material are not synonymous with harmful or detrimental (Ladics et al., 2015). Insertion of transgene into the rice genome may lead to unintended changes which alters endogenous gene expression, subsequently leading to changes in macro- or micro-nutrients, anti-nutritional factors or other essential nutritional component of rice seed.

Dwarfisms of transgenic tomato plants expressing phytoene synthase gene is one of the most striking example of such transgenic plant (Fray et al., 1995). However, such abnormal incidents are found significantly higher in number in traditional breeding and sensible regulation of transgenic crops based on lessons from plant breeding, biotechnology and genomics may help in expediting better crop selection (Bradford et al., 2005). Hence, it is important to study the regulation of metabolic process of carotenoids pathways in Golden rice and select an event with no pleiotropic effect of the alteration which have the potential to save millions of impoverished fellow humans from needless sufferings and death (Alberts et al., 2013).

The regulation of carotenoid biosynthesis is a complex metabolic process (Farré et al., 2013). Therefore, a comprehensive understanding of metabolic regulation and biochemical interactions among the metabolites becomes important for high content carotenoid crop. With the rapid development of GM crops, assessing their bio-safety has also become increasingly important. In addition, the genetic modification of specific pathways may also alter the other closely related pathways. In general, safety assessments mainly focus on detecting any random protein expression or unintended biochemical component in the GM organism due to integration of a foreign gene. The intended and unintended (unexpected) differences between GM and non-GM crops are further assessed to elucidate their potential impact not only on the nutritional quality of the crops but also on human and animal health. Transgenic alterations in GM organisms are monitored by various techniques such as transcriptomic, proteomic, and metabolomic profiling (Decourcelle et al., 2015). The combined proteomic study and metabolomic analysis is one of the most indispensable tools for understanding pleiotropic effect of the molecular alteration and adaptation for biosynthesis of carotenoids.

To combat VAD-induced mortality and morbidity, β-carotene-enriched golden rice has been developed for natural provitamin A supplementation. To develop golden rice, the phytoene synthase (*psy*) of daffodil (*Narcissus pseudonarcissus*) and phytoene desaturase (*crtI*) of pathogenic bacteria (*Erwinia uredovora*) genes were introduced and overexpressed in the endosperm of two popular Asian rice cultivars, IR64 and BR29 using endosperm specific promoter (Datta et al., 2006). Whereas, HPLC analysis revealed that the highest level of carotenoid was accumulated in the endosperm of homozygous transgenic BR29 line (SKBR-244) compared to transgenic progenies of IR64 (SK64-560) in polished grains. Therefore, the present study was aimed to elucidate the metabolic adaptation process of one transgenic golden rice line of BR29 (homozygous SKBR-244 line) due to higher accumulation of carotenoids compared to transgenic IR64 line as a preliminary study.

## Materials and methods

### Rice sample

In our previous study, transgenic golden rice was developed by manipulation of phytoene synthase (*psy*) and phytoene desaturase (*crtI*) gene in the genome of two popular Asian rice cultivars, IR64 and BR29 (Datta et al., 2006). The integration of the transgene was confirmed by PCR analysis using gene specific primers. Furthermore, stable integration of transgene gene in the genome of PCR positive plants were confirmed by Southern blot analysis and followed by segregation analysis. Finally, high carotenoids transgenic line was selected on the basis of carotenoids level by HPLC analysis. The HPLC analysis showed that the highest level of carotenoid was obtained in progenies of transgenic BR29 line (SKBR-244) compared to transgenic progenies of IR64 (SK64-560) in polished grains. Moreover, SK64-560 was not homozygous transgenic line. Therefore, homozygous transgenic T4 progeny of BR29 (SKBR-244) was considered for present study.

The homozygous transgenic golden rice line (SKBR-244) expressing *Psy* and *crtI* gene and non-transgenic counterpart (BR 29) were grown in green house condition at 16/8 h photoperiods with a temperature setting of 35/30°C (day/night) and a light intensity of 2000 lux to obtain rice seeds for proteomics and metabolomics study (Datta et al., 2006). The rice seeds were collected and air-dried at room temperature for adjusting the moisture content to 12–14% before storage at 4°C. The mature rice seeds were dehulled by THU-35C dehuller (Satake, Japan). The de-husked brown rice seeds were homogenized by grinding to fine powder before analysis.

### Genomic PCR and RT-PCR analysis

Genomic DNA was isolated from transgenic and non-transgenic rice seed following the protocol as reported by Dellaporta et al. (1983). The PCR amplification was carried out using *Psy and crtI* gene specific primers. For RT-PCR analysis, RNA was isolated from rice seed (Meng and Feldman, 2010) and cDNA was synthesized from 2.0 μg total RNA using Fermentus cDNA synthesis kit. Twenty microliter PCR reactions were performed using Phusion High-Fidelity DNA Polymerase (Thermo Scientific), 4.0 μl 5X Phusion HF Buffer, 0.5 μl 10 mM PCR dNTPs mix and 0.5 μl each of 10 μM primer and 50 ng DNA for genomic PCR/ 100 ng cDNA for RT-PCR. The thermal cycler protocol was 98°C for 30 s, 35 cycles of 98°C for 10 s, 56°C for 30 s, 72°C for 30 s and a final 10-min extension at 72°C. In this study, β*-tubulin* was used as internal control. All primer sequences used in the study have been provided in Table S1.

### Estimation of carotenoids by HPLC

Carotenoids were extracted from rice seed using 1 ml of NaCl (200 g/L) and 1 ml of 1:1 cyclohexane and ethyl acetate solvent followed by centrifugation at 2000 rpm for 10 min (Eppendorf centrifuge 5415R, Germany). The upper phase was collected and the extraction process was repeated until the elimination of yellow color from rice sample. The extract was dried in the speedvac system (Eyela, Japan) and dissolved in 50 μl 1:1 cyclohexane and ethyl acetate mixture. The carotenoids were estimated by HPLC (Waters, USA) with a multi-solvent delivery system, provided with UV-VIS detector with Empower 3 Chromatography software to acquire and process spectral and chromatographic data. Carotenoids were separated with YMC C30 column using methanol, tertiary butyl methyl ether and water mixture (81:15:4–6:90: 4) (Sander et al., 1994). Analysis of each sample was performed in triplicate.

### Protein extraction and two-dimension gel electrophoresis (2-DE)

Total protein of rice seed (2.0 g) was isolated by phenol extraction method according to Paul et al. (2015). The total protein concentration was measured using Bradford reagent (Sigma, USA) and quality of protein was checked by running in 1D SDS-PAGE. 700 μg of total protein was used for rehydration on 17 cm IPG strip (pH 4–7, Bio-Rad, USA) and isoelectric focussing was performed on the IEF Cell (Bio-Rad, USA) using following condition: 250 V linear for 30 min, 10,000 V linear for 4 h, 10,000 V for 43,000 Vh, 1000 V for 5 min. For second-dimension polyacrylamide gel electrophoresis separation, IPG strips were run onto 12.5% SDS-PAGE gels and proteins were stained with Coomassie Blue G-250 (Sigma, Barcelona, Spain). After staining, the gel picture was captured by Calibrated Imaging Densitometer (Bio-Rad, GS-800) and analyzed by PDQuest Software, version 8.0 (Bio-Rad, USA). Three biologically independent gels were prepared for transgenic and control. All protein spots of gel were detected by matching the spots to the corresponding spots of master gel (reference gel) and normalized each spot density against the whole gel densities. The percentage volume of each spot was averaged for three replicate. Statistical analysis (*t*-test) was conducted to determine the significant differences between the two groups (WT and transgenic). The molecular weight (Mw) and isoelectric point (pI) of each identified spot were measured by comparison with known standard protein marker. Student's *t*- test was performed for statistical analysis and protein spots above 2.0-fold for up-regulation and below 0.5-fold for down-regulation were considered as differentially expressed proteins. The spots were excised from the gel manually and subjected to trypsin digestion using *in-vitro* trypsin digestion kit (Pierce, USA) and peptide identification was done with MALDI-TOF-MS/MS analyzer (Bruker Daltonics, Germany). Spectra of peptides were collected with the Flex Control software and data analysis was carried out using the software Flex Analysis 3.4. The proteins were searched using the MASCOT program (Matrix Science, London, England) and identified by NCBI nr protein sequence database (National Center for Biotechnology Information, Bethesda, MD, USA) using a MOWSE algorithm.

The data was searched against NCBInr database using following parameters: taxonomy: *Oryza sativa* (25805290 sequences); cleavage specificity: trypsin with one missed cleavages allowed; mass tolerance of 100 ppm for precursor ions and a tolerance of 0.7 Da for the fragment ions; allowed modifications: carbamidomethyl (fixed), oxidation of methionine (variable); cleavage by trypsin: cuts C-term side of KR unless the next residue is P. According to MASCOT probability analysis, only significant hits (*P* < 0.05) were considered. The percentage of the sequence coverage, and number of matched peptides and their length corresponding to four and at least five amino acids, respectively.

### Amino acid analysis by AccQ-Tag method

All amino acids were analyzed following the standard procedure of AccQ-Tag (Waters, USA) method as previously described by Gayen et al. (2014). Twenty milligram of rice powder was digested with 2 ml of 6 N HCl containing 0.1% phenol at 110°C temperature for 16 h in the closed glass vial. The hydrolysed sample was passed through 0.22 μm syringe filter. Equal volume of the clear extract was neutralized with equal volume of 6 N NaOH. The amino acids were derivatized by AccQ-Fluor reagent at 55°C for 10 min according to manufacture protocol (Waters, USA). The amino acids were separated by AccQ-Taq Column (150 × 3.9 mm) on a Waters 515 HPLC pump system attached to a Waters 2996 fluorescence detector. Amino acids analysis was performed in triplicate for each sample.

### Mineral analysis

Minerals concentration of rice seed was measured by atomic absorption spectroscopy (AAnalyst200, Perkin Elmer, USA) using the method as reported by Gayen et al. (2013). Transgenic and non-transgenic brown rice seeds about 2.0 g each was ignited in a muffle furnace at 550–600°C for 10 h. The ash of the rice samples was cooled to room temperature and dissolved in 0.2 N HCl solution. The respective metal ions were measured by the hollow-cathode lamp (HCL). Mineral content was analyzed in triplicate for transgenic and control.

### Metabolomics study

The metabolite extraction and derivatization was carried out following the protocol of Roessner et al. (2000). Approximately, 300 mg of rice seed powder was homogenized in 1.4 ml of methanol (100%) and 50 μl of internal standard (2 mg/ ml of sorbitol in water). The mixture was vortexed and incubated at 70°C for 15 min followed by centrifugation for 10 min at 2200 g. The methanol/ water fraction (1000 μl) was dried in speed vac and stored in −20°C. The dried sample was dissolved in 40 μl of methoxyamine hydrochloride (20 mg/ ml in pyridine) solution and incubated at 37°C for 90 min. For trimethylsilylation, 60 μl MSTFA was added and incubated at 37°C for 30 min. One microliter of sample was injected in GC-MS (Shimadzu QP-2010) using auto sampler. Metabolites were analyzed in triplicate for each sample.

### Reducing sugar estimation

Reducing sugars of rice seeds was measured by following Miller (1972). The rice seed were powdered by mortar and pestle and extracted with 80% ethanol. The extracted sample was boiled with DNS followed by 40% Rochelle reagent. After cooling the reaction mixture, absorbance was measured at 510 nm and expressed as mg/g DW tissue.

### Statistical analysis

Statistical significance was analyzed for all experiments by the unpaired student's *t*-test method using GraphPad Prism 5. *P* < 0.05 was considered to be statistically significant, and the results are expressed as mean ± SE (*n* = 3).

## Results

The carotenoid pathway in rice was genetically modified by the introduction of exogenous *psy* and *crtI* along with an endosperm-specific glutelin promoter (Datta et al., 2006). In the present study, the proteome profiles and metabolites of transgenic golden rice line (T4 progeny of SKBR-244) with high carotenoid content were compared with its non-transgenic counterpart BR29 rice. The presence and expression of both the genes were confirmed by PCR and RT-PCR analysis using gene specific primers before carrying the proteomic and metabolite analysis (Figure S1 and Table S1).

### Proteomics study

**Figure 1:**
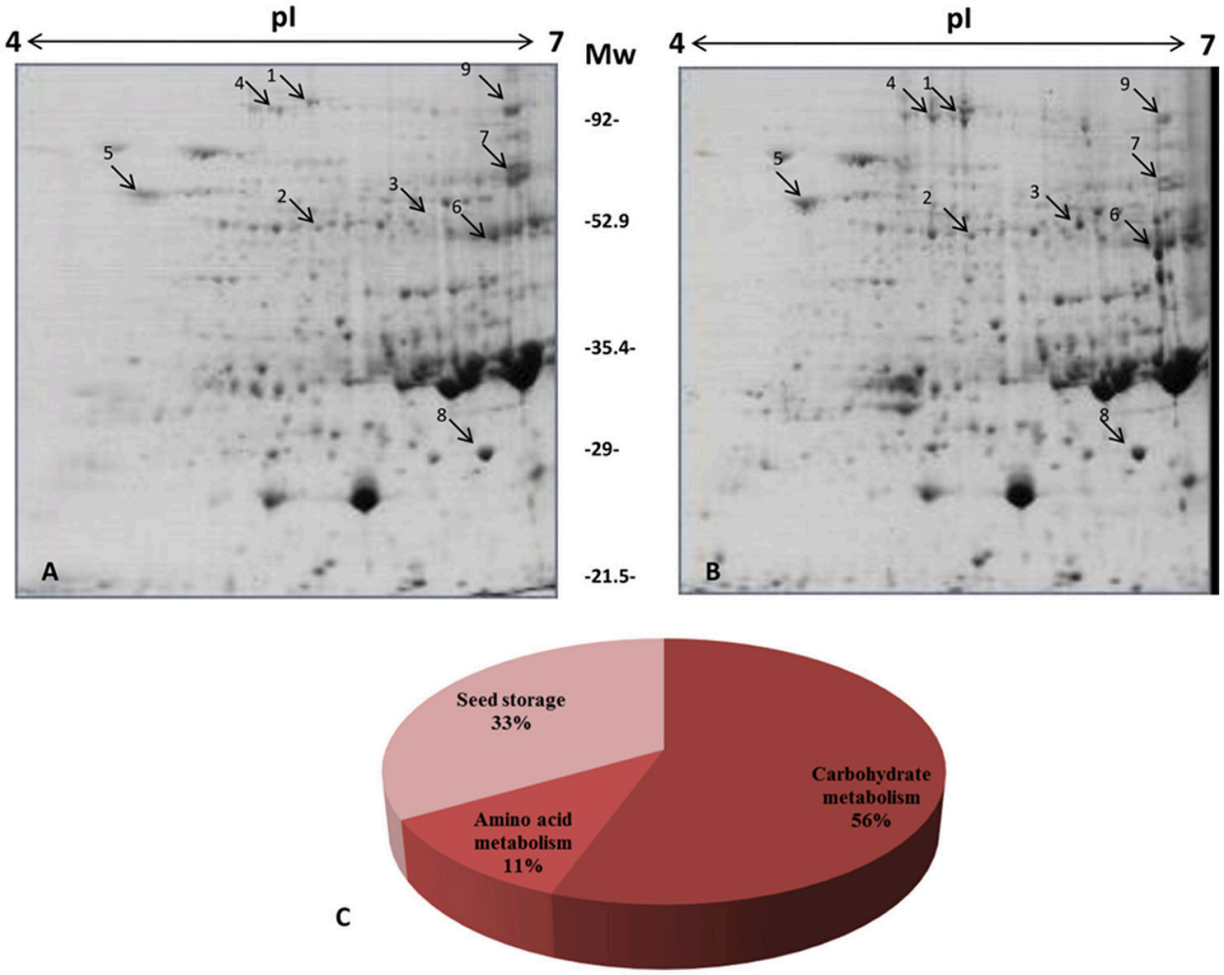
**Comparative protein profile of transgenic and control rice seeds by two-dimensional gel electrophoresis (2-DE)**. Seed proteins (700 μg) were separated on pH 4–7, IPG strips and then separated on 12% SDS-polyacrylamide gels. Proteins were visualized by colloidal staining. **(A)** Control **(B)** Transgenic rice **(C)** Distribution of protein according to functional pathways. The information of differentially expressed proteins has been provided in Table 1.

We conducted a comparative proteome profiling of transgenic golden rice seeds and control rice seeds using 2D gel electrophoresis (Figure 1). The PDQuest analysis identified nine differentially expressed proteins (DEPs). Accumulated protein spots of >2.0-fold and <0.5-fold were considered as DEPs. In addition, the PDQuest analysis revealed that six proteins were up-regulated and three proteins were down-regulated (Table 1).

**Table 1:** **Differentially expressed proteins of transgenic golden rice and control rice seed**.

**Spot no**	**Accession no^a^**	**score**	**Coverage (%)**	**Peptideno**	**Theoretical pI/MW**	**Experimental pI/MW**	**Protein**	**Fold change^b^**
**CARBOHYDRATE METABOLISM**								
1	gi|262345489	711	35	32	5.44/102.57	5.54/102.	Pullulanase	3.36
2	gi|88866516	439	56	24	5.43/51.65	5.58/53.0	UDP-glucose pyrophosphorylase (EC: 2.7.7.9)	3.46
3	gi|115476014	821	62	27	5.87/52.9	6.25/57.84	Glucose-1-phosphate adenylyltransferase. (EC: 2.7.7.27)	2.5
4	gi|218196777	588	35 57	33	5.37/93.6	5.4/95.4	PPDK (EC: 2.7.9.1)	3.06
5	gi|115469436	396		19	6.19/42.27	6.75/48.03	Phosphoglycerate kinase (EC: 2.7.2.3)	2.9
**AMINO ACID METABOLISM**								
6	gi|62546209	792	50	25	4.95/56.85	4.6/58.0	Prolyl hydroxylase (EC: 1.14.11.2)	3.05
**SEED STORAGE PROTEIN**								
7	gi|125588221	193	45	15	6.99/49.98	6.91/60.5	hypothetical protein OsJ_12925	0.29
8	gi|13310891	88	24	12	9.14/51.59	6.6/28.6	Hypothetical protein	0.32
9	gi|218193892	258	37	16	6.99/52.06	6.9/88.51	hypothetical protein OsI_13867	0.14

a protein accession number against NCBInr data base;

b Average fold change of three replicate gel.

After image analysis, the DEPs were digested with trypsin, and subsequently, mass spectrometry analysis was performed using MALDI-ToF MS/MS. The fragmented mass spectra of the protein spots were searched against a non-redundant database in NCBI using MASCOT. The average fold change of the identified protein species in transgenic crops represents the ratio of spot intensity change compared with its non-transgenic counterpart (Table 1). Proteins having more than two peptides with a significant change in Mascot score (*p* < 0.05) were studied. Additional information about proteomic analysis can be found in Table S2.

GO-enrichment analysis for the identified DEPs was performed using the Blast2GO software. The identified proteins were grouped in the following three functional categories: carbohydrate metabolism, amino acid metabolism and seed storage proteins. Among the identified DEPs, pullulanase, UDP-glucose pyrophosphorylase (EC: 2.7.7.9), glucose-1-phosphate adenylyltransferase (EC: 2.7.7.27) and pyruvate phosphate dikinase (PPDK) were categorized under carbohydrate metabolism. The relevant enzymes of carbohydrate metabolism, pullulanase and UDP-glucose pyrophosphorylase (EC: 2.7.7.9), were enhanced by 3.36- and 3.46-fold, respectively, in transgenic rice compared with the non-transgenic control seeds. Furthermore, glucose-1-phosphate adenylyltransferase (EC: 2.7.7.27) level was also increased in transgenic rice seeds by 2.5-fold. Pyruvate is one of the main components of the carotenoid pathway. Upregulation of the important proteins, such as pyruvate phosphate dikinase (PPDK) (EC: 2.7.9.1) and prolyl hydroxylase (EC: 1.14.11.2), were also found in transgenic rice seeds as shown in the Table 1. The enhanced expression of pyruvate phosphate dikinase (PPDK) increases pyruvate biosynthesis in seeds. The photosynthesis-related protein (EC: 2.7.2.3) was found to be upregulated (2.9-fold) in transgenic rice seeds compared to control seeds. In addition, proteins such as OsJ_12925, gi|13310891 and OsI_13867, which are associated with seed storage activity, were found to be down-regulated in the transgenic seeds.

### Mineral and amino acid content

Nutritional values of the transgenic high carotenoid rice were analyzed with respect to non-transgenic BR29 rice.

The mineral content of rice seeds was analyzed by atomic absorption spectroscopy (Table 2). Compared to non-transgenic rice, transgenic rice had slightly higher concentrations of sodium (3.4%), copper (9.4%), and zinc (5.0%). Moreover, all the data were within the reference range and the variation between transgenic and non-transgenic rice was not statistically significant (Table 2).

**Table 2:** **Mineral composition of golden rice and non-transgenic BR29 rice seeds (brown)**.

**Components (mg/100 g)**	**Golden rice**	**BR29**	**Ref. Range^a^**
Sodium	4.75 ± 0.23	4.59 ± 0.30	2–40
Potassium	285.33 ± 6.25	290.75 ± 12.80	70–320
Copper	0.197 ± 0.01	0.18 ± 0.01	0.1–0.7
Magnesium	104.73 ± 2.93	106.90 ± 00	20–170
Manganese	1.43 ± 0.04	1.50 ± 0.01	0.2–4.2
Iron	0.97 ± 0.16	1.01 ± 0.44	0.2–6.0
Zinc	1.67 ± 0.02	1.59 ± 0.01	0.7–3.3

a Source: OECD (2004).

Data are means ± SEs of three replicates.

**Figure 2:**
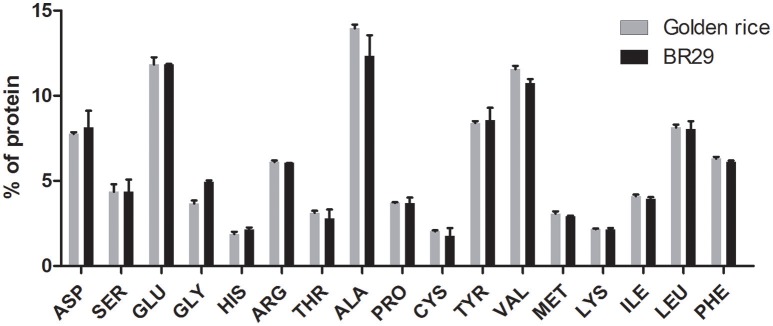
**Amino acids composition of golden rice and non-transgenic BR 29 rice seeds**. Rice seed protein was hydrolysed by 6 N HCl at 110°C temperature. The amino acids were derivatized using AccQ-Fluor reagent and separated by AccQ-Taq Column (150 × 3.9 mm) and detected by fluorescence detector. Amino acid content was measured based on amount of amino acids standard. Values are represented by mean of three replicates ± standard error (SE). Statistical significance difference between transgenic and control was determined using unpaired *t*-test (*p* < 0.05).

In this study, 15 individual amino acids found in rice seeds were analyzed using an automated amino acid analyzer (Water, USA). The amino acids are expressed as percentage (%) relative to total protein (Figure 2). The amino acid profiles of transgenic rice seeds were very similar to that of non-transgenic rice seeds. The glycine content in transgenic rice seeds (3.67%) was lower than that of BR29 (4.93%), whereas the alanine (13.0%), valine (7.5%), cystine (15.34%), methionine (5.8%), and threonine (11.4%) contents in transgenic rice seeds were slightly higher than that of BR29.

### GC-MS based metabolomics analysis

**Figure 3:**
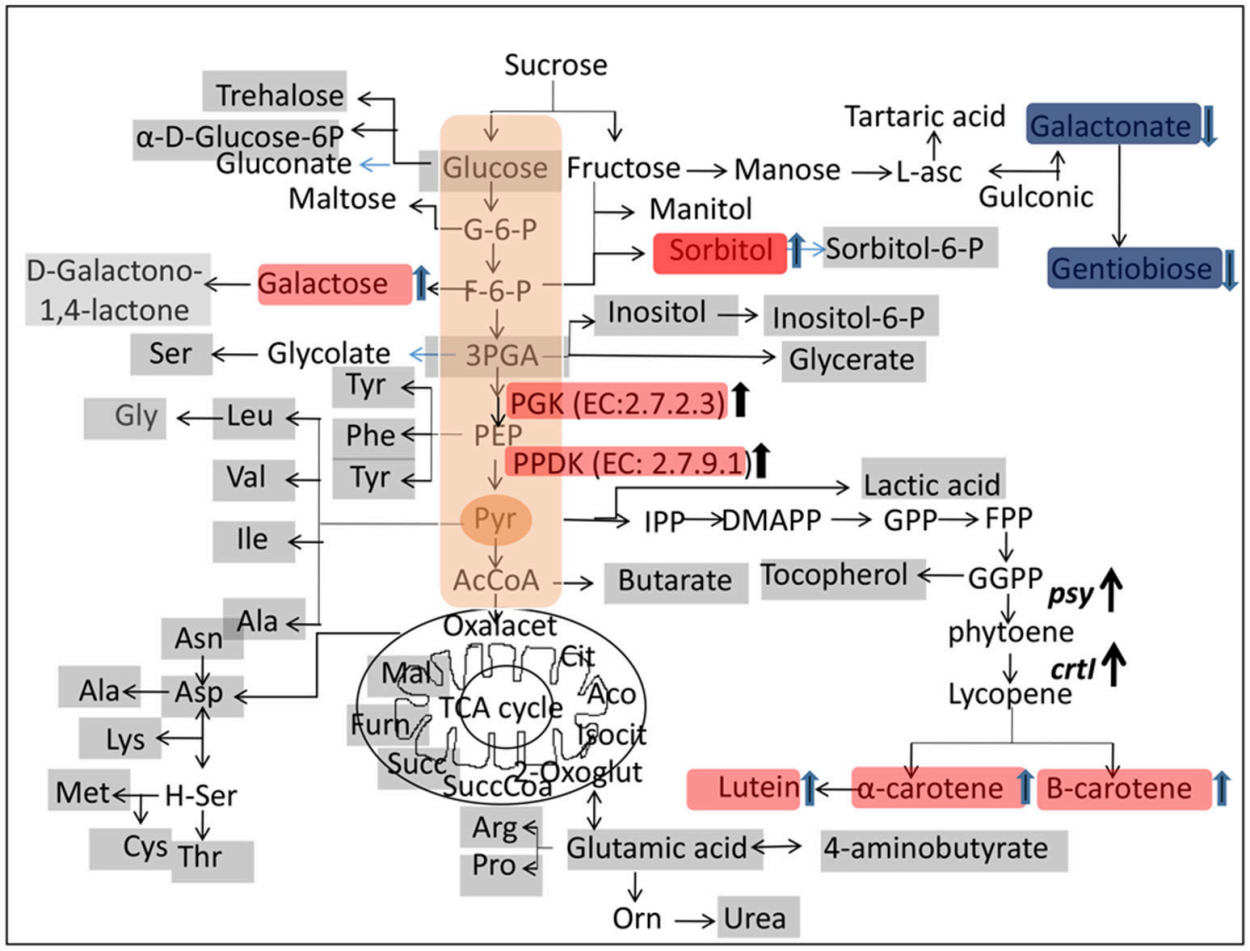
**Metabolic and proteomic changes in transgenic and control rice seed**. The metabolites of rice seeds were analyzed by GC-MS and changes are demonstrated by diagram. The metabolites marked on gray background were detected in this study. The compounds marked by red background were increased significantly (*P* < 0.05) in transgenic rice seed compared to control. The compound indicated by blue background were decreased statistically (*P* < 0.05) in transgenic rice seed compared to control. Abbreviations: G-6-P, glucose 6-phosphate; F-6-P, fructose 6-phosphate; PGA, glycerol-3-phosphate; GPP, geranyl pyrophosphate; FPP, farnesyl pyrophosphate; GGPP, geranylgeranyl pyrophosphate; DMAPP, dimethylallyl diphosphate; IPP, isopentenyl pyrophosphate; PPDK, Pyruvate phosphate dikinase; PGK, Phosphoglycerate kinase.

A metabolite map was constructed to elucidate the pathway that was affected by genetic manipulation. The metabolite changes in transgenic rice were statistically significant compared to non-transgenic rice (*p* < 0.05) (Table 3). Metabolites marked with red background indicate a significant increase in transgenic rice whereas those in blue indicate a significant decrease compared to the control (Figure 3). Among the metabolites of the carbohydrate pathways, galactonate, gentiobiose, and glycerol-3-phosphate were decreased in transgenic rice seeds as compared to BR29 rice seeds, whereas glyceric acid, fructo-furanose, galactose and sorbitol were found in higher concentration in transgenic rice seed.

**Table 3:** **Metabolites of rice seeds identified by GC-MS**.

**Peak number**	**RT (min)**	**Components**	**Mean ratio Golden rice / WTa**	***p*-value**	**Pathway**	**KEGG compound**
1.	6.52	Ethylene glycol	0.99	0.72	Others	C01380
2.	6.73	3-Propylnorleucine	0.96	0.56	Others	NA
3.	6.86	Propylene glycol	1.08	0.45	Others	C00583
4.	8.43	Lactic acid	1.06	0.63	Carbohydrate	C00186
5.	12.20	Malonic acid	0.74	0.055	Carbohydrate	C00383
6.	12.31	Butanoic acid	1.05	0.79	Carbohydrate	C00246
7.	12.46	Acetamide	0.97	0.72	Others	C02693
8.	13.38	Urea	0.99	0.93	Others	C00086
9.	13.88	Ethanolamine	0.75	0.06	Others	C00346
10.	14.25	α-D-Glucose-6P	0.88	0.068	Carbohydrate	C00688
11.	15.14	Succinic acid	1.04	0.69	Carbohydrate	C00042
12.	15.76	Glyceric acid	1.45	0.0003	Carbohydrate	C00258
13.	16.04	Fumaric acid	0.89	0.52	Carbohydrate	C00142
14.	18.20	D-Galactono-1,4-lactone	1.11	0.38	Carbohydrate	C03383
15.	19.86	Malic acid	1.03	0.84	Carbohydrate	COO149
16.	20.74	GABA	1.13	0.69	Amino acid	C00334
17.	22.98	Glutamic acid	0.89	0.19	Amino acid	C00025
18.	26.36	Glycerol-3-phosphate	0.84	0.018	Lipid	C00093
19.	27.29	Fructo furanose	2.74	0.03	Carbohydrate	NA
20.	27.53	Biphenyl -4 carboxylic acid	1.03	0.69	Carbohydrate	NA
21.	28.68	EICOSANE	0.97	0.24	Lipid	NA
22.	29.34	Galactopyranose / galactose	2.14	0.025	Carbohydrate	C00124
23.	29.86	D-Sorbitol	2.74	0.013	Carbohydrate	C00794
24.	30.92	D-Glucose	1.12	0.11	Carbohydrate	C00031
25.	31.19	Galactonic acid	0.61	0.0014	Carbohydrate	C00880
26.	31.60	Hexadecanoic acid	0.77	0.08	Carbohydrate	C00249
27.	32.64	Ferulic acid	0.72	0.08	Secondary metabolite	C01494
28.	32.89	Myo-Inositol	0.97	0.60	Carbohydrate	C00137
29.	34.74	OELSAEURE	0.88	0.60	Lipid	NA
30.	37.39	9-OCTADECENAMIDE	0.92	0.60	Lipid	C19670
31.	37.48	INOSITOL-3-p	1.17	0.25	Carbohydrate	C04006
32.	38.65	D-MYO-INOSITOL	1.04	0.21	Carbohydrate	C000137
33.	40.08	Gentiobiose	0.65	0.002	Carbohydrate	C08240
34.	40.43	Cellobiose	0.80	0.007	Carbohydrate	C00185
35.	43.17	Adenosine	1.04	0.83	Nucleic acids	C00212
36.	45.25	Octadecenoic acid	0.71	0.10	Lipid	C00712
37.	45.74	Trehalose	1.13	0.10	Carbohydrate	C01083
38.	47.83	D-Glucuronic acid	1.58	0.005	Carbohydrate	C00257
39.	49.34	Tocopherol-.gamma	1.30	0.21	CPGEC	C02483

The column of Golden rice/ BR 29 shows the ratios of relative metabolite levels between transgenic and control. Red and green shaded cells indicate that the mean values are significantly higher and lower in transgenic golden rice and control BR 29, respectively. P-value indicates the significance of difference of the relative metabolite levels between Golden rice and BR 29. ^a^Average ratio of three independent analysis.

### Carotenoids analysis

**Figure 4:**
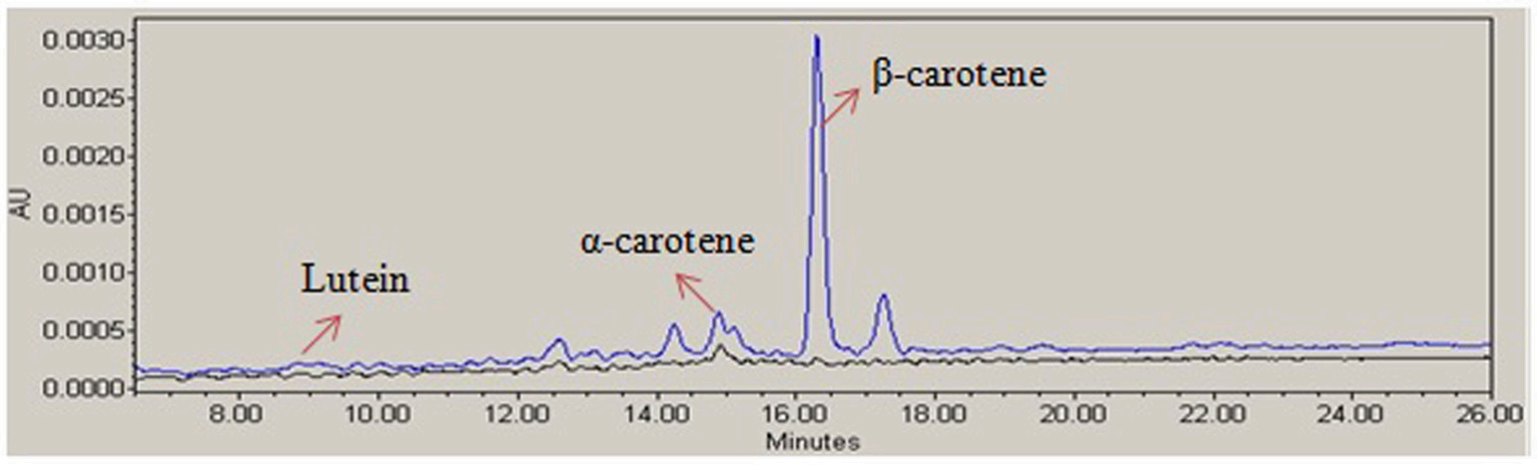
**Analysis of carotenoids of rice seed**. A representative high-performance liquid chromatography (HPLC) profile of the carotenoids extract of transgenic golden rice (blue) and non-transgenic BR29 (black) showing β-carotene, α-carotene, and lutein peak (showed by arrows).

The carotenoid biosynthesis was enhanced in transgenic golden rice after genetic manipulation of endosperm specific pathways. The carotenoid content of rice seeds was measured by HPLC analysis (Figure 4). Among the carotenoids, β-carotene was found at higher concentrations in golden rice. The HPLC analysis showed that the transgenic rice contained about 4.3 μg/g of β-carotene, whereas the contents of α-carotene and lutein were 0.59 μg/g and 0.17 μg/g respectively. However, the non-transgenic BR 29 seed contained very negligible amount of carotenoids.

## Discussion

Rice is a staple food crop for human consumption. In plants, carotenoids are synthesized in all photosynthetic green tissues. However, carotenoids do not accumulate in the rice seed endosperm. In this context to overcome VAD, by supplementing vitamin A by natural source, a transgenic rice variety expressing carotenoids has been developed by the introduction of *crtI* and *psy* in the genome of *indica* rice BR29 (Datta et al., 2006). However, carotenoid biosynthesis is complex process and is restricted to specific tissues (Lima et al., 2005), where isoprenoid precursor plays a major role.

Since genetic manipulation of an organism also affects other relevant pathways, this study carried out proteomic and metabolite analyses using transgenic high carotenoid rice seeds to comprehend the impact of transgene expression on the core metabolic pathways of the rice endosperm. The two key genes of carotenoid pathway were introduced into the genome of BR29 and IR64 for the biosynthesis of carotenoids in the rice endosperm. The expression level of carotenoids of IR64 line was very less, whereas SKBR-244 showed a higher level of expression. Therefore, based on the carotenoids accumulation in transgenic rice seed, we proceed further study with SKBR-244 line. Additionally, SK64-560 was not homozygous transgenic line as shown in our previous study (Datta et al., 2006).

Rice is a good source of minerals that are required for normal metabolic activities (Rayan and Abbott, 2015). The mineral content of transgenic rice seeds was comparable to non-transgenic rice seed.

Amino acids, important building blocks of protein, play a significant role in physiological processes. Despite small differences in amino acid level, no statistical significant difference was observed among other amino acids as shown in previous study (Lepping et al., 2013).

HPLC analysis showed that carotenoid content was enhanced in transgenic golden rice compared to non-transgenic rice due to integration *Psy* and *crtI* genes. Phytoene synthase is the first committed enzyme in carotenoid biosynthesis and it is also a rate-limiting enzyme for carotenoid production in the endosperm (Farré et al., 2013). The phytoene synthase is the first enzymatic step which produces geranylgeranyl diphosphate (GGPP) (Bai et al., 2016). Reverse transcription polymerase chain reaction (RT-PCR) indicated that the mRNA transcription of *psy* and *crtI* gene were present in transgenic rice seed whereas the expression was absent in the non-transgenic rice seed.

Metabolomics is an important tool for biological system for understanding molecular mechanism (Fukushima and Kusano, 2013; Yadav et al., 2015). To investigate the metabolite alteration due to genetic manipulation in the rice samples, metabolite profiling was performed by GC-MS analysis. The protocol followed was similar to that reported for maize (Decourcelle et al., 2015), potato (Shepherd et al., 2015), and rice (Kusano et al., 2011).

The metabolite profiling demonstrated that metabolites related to carbohydrate metabolism were altered due to genetic manipulations in order to adjust the metabolic flux required for carotenoids biosynthesis. In order to confirm the hypothesis, alteration of carbohydrate in rice seeds of the different independent transgenic line of BR29 and IR64 (SKBR-244, SKBR-216, and SK64-560) was also checked by estimation of reducing sugar by DNS method. Transgenic rice seeds showed high accumulation of reducing sugar compared to the corresponding control (Figure S2). The result clearly indicated the similar trend of altered carbohydrate metabolites due to overexpression of the phytoene synthase (*psy*) and phytoene desaturase (*crtI*) gene. The GC-MS analysis revealed that glycerol-3-phosphate was down regulated slightly in transgenic rice seed due to increased expression of PPDK enzyme (EC: 2.7.9.1). However, the levels of fructo furanose, galactose, D-Sorbitol and D-Glucuronic acid were elevated in the GM lines compared with the WT rice line. The comprehensive metabolites analysis of transgenic golden rice showed that no major changes were detected in others metabolites.

A comparative proteomic study of GM organisms is an indispensable tool for safety assessment of GM crops. Sometimes, integration of a foreign gene may lead to mutation, pleiotropic effect, or inactivation of endogenous genes, which can alter other metabolic pathways. Currently, proteomics is widely used to assess GM organisms, evaluating their intended and unintended toxic or nutritionally harmful effects. The proteomic study revealed that pullulanase activity was enhanced by 3-fold in transgenic golden rice compared to non-transgenic rice (Table 1). Pullulanase plays a significant role in debranching the starch molecule and producing a sugar molecule, which takes part in different biochemical pathways including carotenoid biosynthesis. Glucose is the initial precursor of carotenoid biosynthesis (Decourcelle et al., 2015). Plastid is an organelle where carotenoids are synthesized and starch is degraded for maintaining the carbon source (Cao et al., 2015). Moreover, enhanced pullulanase activity in transgenic golden rice leads to the formation of a glucose which might increase the carbon supply of transgenic rice seed as shown in metabolomic analysis.

Furthermore, UDP-glucose pyrophosphorylase (EC: 2.7.7.9) activity was also enhanced by 3.46-fold in transgenic rice compared to non-transgenic rice. UDP-glucose pyrophosphorylase is a sugar-metabolizing enzyme that catalyzes a reversible reaction of UDP-glucose and pyrophosphate from glucose-1-phosphate and UTP (Payyavula et al., 2014). UDP-glucose pyrophosphorylase is a key enzyme of carbohydrate metabolism, which is linked to other multiple metabolic pathways (Chen et al., 2007). Down-regulation of *Ugp1* in rice leads to detrimental effects on plant growth and development (Chen et al., 2007). The over expression of UDP-glucose pyrophosphorylase (EC: 2.7.7.9) in transgenic golden rice seeds predominantly catalyzes sucrose biosynthesis, serving as the precursor of carotenoid biosynthesis. Furthermore, UDP-glucose is converted to glucuronic acid, as shown by the KEGG pathway. The metabolomic analysis also reflected that glucuronic acid was up-regulated by 1.58-fold in transgenic rice seeds. Therefore, it is hypothesized that increased levels of glucuronic acid are directly proportional to higher concentrations of UDP-glucose pyrophosphorylase in transgenic rice compared to non-transgenic rice. In addition, it can be assumed that the expression of glucose-1-phosphate adenylyltransferase, up-regulated in transgenic rice seeds may increase starch biosynthesis.

PPDK (EC: 2.7.9.1) is a key enzyme of the pyruvate metabolism pathway that catalyzes the formation of pyruvate from phosphoenolpyruvate in developing rice seeds (Kang et al., 2005; Chastain et al., 2006, 2011; Hennen-Bierwagen et al., 2009). In all plants, PPDK is located in both the cytoplasmic and plastid compartments (Chastain and Chollet, 2003). However, PPDK is also an abundant glycolytic enzyme found in the rice seed embryo and predominantly catalyzes pyruvate biosynthesis, an important precursor of the non-mevalonate pathway condensing pyruvate and glyceraldehyde-3-phosphate for starch, fatty acid, protein, and amino acid biosynthesis (Chastain et al., 2011). One possible explanation is that higher PPDK activity was found to deplete the pool of glyceraldehyde-3-phosphate in transgenic rice seeds. Thus, the elevation of PPDK expression in transgenic rice seeds might be one of the factors that potentially trigger carotenoid biosynthesis. HPLC analysis clearly demonstrated that β-carotene, α-carotene and lutein contents of transgenic rice were enhanced due to transgene integration (Figure 4). In addition, tocopherol was elevated by 1.30-fold in transgenic rice seeds in comparison with the non-transgenic rice seeds. Both carotenoids and tocopherol are produced from the common precursor geranylgeranyl pyrophosphate, which is produced from pyruvate (Figure 3). The amino acids like alanine, cysteine, valine, threonine produces from pyruvate and oxaloacetate as shown in Figure 3 (Yamakawa and Hakata, 2010). Further, amino acid contents, especially that of alanine, valine, cysteine, and threonine were also found to be slightly higher in transgenic rice and over accumulation of pyruvate due to higher PPDK activity may be the reason. The accumulation of amino acids might contribute significant role in grain quality. Therefore, this study demonstrates that pyruvate biosynthesis may be the key for nutritional improvement in rice seeds.

Prolyl hydroxylase plays a significant role in the synthesis and deposition of storage proteins. It has been reported that prolyl hydroxylase has chaperone activity and inhibits the aggregation of misfolded proteins that play vital roles in the maturation of secreted plasma membrane and storage proteins (Kim et al., 2012). Prolyl hydroxylase directly provide disulfides to substrate proteins via thiol-disulfide exchange reactions. Prolyl hydroxylase activity in transgenic golden rice was found to be enhanced. In addition, phosphoglycerate kinase (Os06g066820) (EC: 2.7.2.3), an important enzyme of the glycolysis pathway, was found to be up-regulated in transgenic rice by 2.9-fold compared to the non-transgenic rice. Enhanced expression of phosphoglycerate kinase (EC: 2.7.2.3) leads to increase pyruvate pool which is an essential building block for the DXS pathway for carotenoids biosynthesis (Decourcelle et al., 2015).

Precursor of carotenoids biosynthesis originates from sugar metabolism (Decourcelle et al., 2015). The pyruvate and glyceraldehyde-3-phosphate are the key precursors for carotenoids biosynthesis (Kim et al., 2010). However, glyceraldehyde-3-phosphate was found in lower concentration in transgenic rice (Figure 3). This may be due to higher expression of PGK and PPDK enzymes as shown by proteomics study. The expressions of seed storage protein gi|115464709, gi|125588221 and gi|218193892 were decreased in transgenic rice seeds compared to non-transgenic rice seeds. Rice seed contains about 7–15% storage protein of dry weight and maintain a constant level of nitrogen source in order to germinate. Alteration of seed storage protein (SSP) is compensated by an increase or decrease other endogenous storage protein by rebalancing mechanisms to conserve constant levels of total seed protein (Takaiwa, 2013). Expression of recombinant proteins suppress the endogenous protein level in order to compensate. In this study, transgenic golden rice was developed by over expression of exogenous *psy* and *crtI* gene under the control of endosperm specific glutelin promoter. Therefore, the expression of foreign protein decreased the SSP protein of rice seed embryo to balance the total protein. Yasuda et al. (2009) showed that overexpression of BiP protein decreased the seed storage protein. In another study, suppression of 7S β-conglycinin protein in soybean seeds leads to increase in seed storage protein 11S glycine (Mori et al., 2004).

Furthermore, the present study also revealed that sorbitol was enhanced in transgenic rice. Similar results were observed in transgenic high carotenoid maize seeds (Decourcelle et al., 2015). Kim et al. (2010) demonstrated that carotenoid biosynthesis is influenced by the carbon source. Therefore, the elevation of fructo furanose and the galactose level in transgenic rice seeds may participate in carotenoid biosynthesis (Table 3).

In the present study, transgenic golden rice was developed by introduction of phytoene synthase (*psy*) and phytoene desaturase (*crtI*) to enhance the carotenoids content of rice endosperm. However, Bai et al. (2016) developed transgenic rice by over expression of endosperm-specific expression of maize (Zea mays) PSY (*ZmPSY*) and Pantoea ananatis CRTI (*PaCRTI*) under the control of the rice glutelin promoter. Additionally, the mini pathways with *Arabidopsis thaliana* 1-deoxy-D-xylulose-5-phosphate synthase (*AtDXS*) and *A. thaliana* ORANGE (*AtOR*) genes were expressed in the rice endosperm under control of endosperm-specific promoter to enhance metabolic flux and sink respectively. Therefore, the study suggested that precursor pool and metabolic sink is also important for engineering of high carotenoids rice seeds (Bai et al., 2014, 2016).

However, the present comparative study indicated that expression of both PPDK and Phosphoglycerate kinase were enhanced in transgenic rice seed to increase pyruvate level which is the precursor of isoprenoid of MEP pathway. The carotenoids level of rice seed may enhance by supplying precursor pool of pyruvate. Therefore, expression of PPDK and Phosphoglycerate kinase may contribute pivotal role in combination of phytoene synthase (*psy*) and phytoene desaturase (*crtI*) for the development of high carotenoids rice seed.

## Conclusions

To understand the metabolic changes and adaptation leading to enhance carotenoid biosynthesis in rice seeds, proteomic, and metabolomic analyses were performed on transgenic golden rice seeds. Carotenoids are synthesized primarily from the precursors derived from the MEP pathway. Pyruvate is the main precursor of MEP pathway. The proteomic analysis clearly showed that expression of PPDK enzyme is increased in transgenic rice seeds, which enhances biosynthesis of pyruvate. The present study showed that expression of the *Psy* and *crtI* genes induces the expression of PPDK enzyme in the rice endosperm of transgenic rice seed. As a result of that carotenoid accumulation is enhanced in rice seeds as compared to non-transgenic controls. As proteomic and metabolomic analyses indicated, maximum metabolic adaptation was observed in carbohydrate metabolism pathways to provide carbon supply for carotenoid biosynthesis of transgenic rice seeds. However, no detrimental effects were observed in both proteome profile and metabolite levels. Besides nutritional assessment, our study demonstrated the deeper insight into the adaptation process of genetically modified golden rice, balancing the expression of enzyme, and metabolites of other pathways for higher carotenoid biosynthesis.

## Author contributions

KD, SD, and DG: Conceived and designed the research; DG, SG, SP, and SS: Conducted experiment and analyzed data; DG: Wrote the paper; KD and SD: Revised the manuscript critically and all authors approved for publication.

## Funding

The study was supported by grants from Department of Biotechnology, Govt. of India (BT/PR12656/COE/34/22/2015), ICAR (DRR/CRP/BIOFORTIFICATION 2015/2976).

### Conflict of interest statement

The authors declare that the research was conducted in the absence of any commercial or financial relationships that could be construed as a potential conflict of interest.
