# Supplementary material for: Metabolic Regulation of Carotenoid-Enriched Golden Rice Line
Source: Front Plant Sci. 2016 Oct 28;7:1622. doi: 10.3389/fpls.2016.01622 (PMC5083848; doi:10.3389/fpls.2016.01622)
Supplement: Supplementary file 1 [file Table1.DOC]

Table S1: Primers used in genomic PCR and qRT PCR analysis for transgenic golden rice

| Gene name | Accession number | Primer sequences | Amplicon (bp) |
| --- | --- | --- | --- |
| *Psy* | X78814 | 5’-TGGTGGTTGCGATATTACGA-3’  5’-ACCTTCCCAGTGAACACGTC-3’ | 979 |
| *crtI* | D90087 | 5’-CGATCCCAGTGCCATTGAAG-3’  5’-CATCCCCTGAACTAATGCGC-3’ | 508 |
| β-tubuline | Os01g59150 | 5’-GGAGTCACATGCTGCCTAAGGTT-3’  5’-TCACTGCCAGCTTACGGAGG-3’ | 64 |
