# Supplementary material for: Metabolic Regulation of Carotenoid-Enriched Golden Rice Line
Source: Front Plant Sci. 2016 Oct 28;7:1622. doi: 10.3389/fpls.2016.01622 (PMC5083848; doi:10.3389/fpls.2016.01622)
Supplement: Supplementary file 3 [file Image1.PDF]

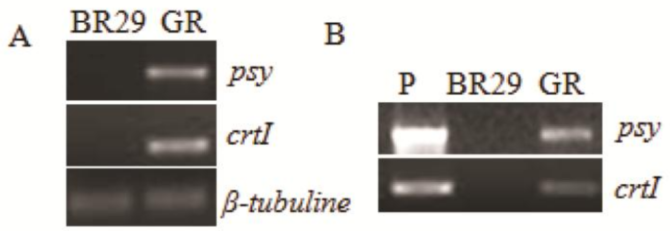

Figure S1: RT-PCR analysis of transgenic high carotenoids rice seeds as compared to control. (A) Expression of *crtI and psy gene. β-tubulin* was used as reference gene. (**B**) PCR confirmation of *crtI* and *psy* gene. P=Positive control plasmid, BR29=Non-transgenic control rice , GR= transgenic golden rice.
