# Supplementary material for: Metabolic Regulation of Carotenoid-Enriched Golden Rice Line
Source: Front Plant Sci. 2016 Oct 28;7:1622. doi: 10.3389/fpls.2016.01622 (PMC5083848; doi:10.3389/fpls.2016.01622)
Supplement: Supplementary file 4 [file Image2.PDF]

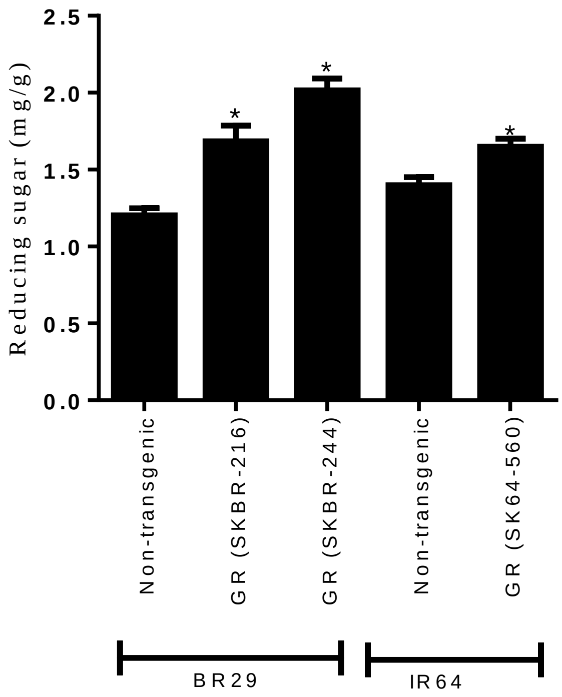

Figure S2: Estimation of reducing sugar of different independent transgenic golden rice (GR) and non-transgenic rice seed. Values are represented by mean of three replicates ± standard error (SE). Statistical significance difference between transgenic and control was determined using unpaired t-test ($p < 0.05$).
